# Instructors' Perceptions of Mostly Seated Exercise Classes: Exploring the Concept of Chair Based Exercise

**DOI:** 10.1155/2016/3241873

**Published:** 2016-11-20

**Authors:** Katie R. Robinson, Tahir Masud, Helen Hawley-Hague

**Affiliations:** ^1^Division of Rehabilitation and Ageing, University of Nottingham, Queen's Medical Centre, Nottingham NG7 2UH, UK; ^2^Bassetlaw Health Partnership, Nottinghamshire Healthcare NHS Foundation Trust, Day Rehabilitation, Retford Primary Care Centre, North Road, Retford DN22 7XF, UK; ^3^Healthcare of Older People, Nottingham University Hospitals NHS Trust, Queen's Medical Centre, Nottingham NG7 2UH, UK; ^4^Nursing, Midwifery and Social Work, The University of Manchester, Jean McFarlane Building, Floor 6, Room 332, Oxford Road, Manchester M13 9PL, UK

## Abstract

Chair based exercise (CBE) can be used to engage older adults unable to take part in standing exercise programmes. Defining and understanding the context of CBE have been acknowledged as a challenge. We explore instructor experiences of delivering mostly seated exercise classes for older people and how this helps us to further understand the concept of CBE. We extracted qualitative data from a cross-sectional survey with 731 exercise instructors. 378 delivered mostly seated classes and 223 of those instructors provided qualitative data. There were 155 instructors who did not provide any qualitative comments. Framework analysis was used and informed by a Delphi consensus study on CBE. Instructors perceived mostly seated classes as predominantly CBE; they defined it as an introductory class that should be offered as part of a continuum of exercise. It was considered suitable for those with limitations and older adults in long-term care and with dementia. Instructors reported CBE used inappropriately for more active older people. Instructors reported observing improvements in mood and cognition and broader social benefits. Instructors' perspectives largely support expert consensus that CBE has an important role in a continuum of exercise. Providers of CBE need to ensure that more challenging exercises are introduced where appropriate. Further research is needed to explore older adults' perceptions of CBE.

## 1. Introduction

Exercise has well known health benefits for older people [1]. Encouraging activity throughout the life course and into older age is a health promotion challenge [2] with many older adults aware of the benefits of exercise but the reported levels of activity remain low [3].

Muscle strengthening and balance training programmes that involve exercising when standing are widely employed in clinical practice and these programmes have been shown to reduce the risk of falls [4] with an associated impact on mortality [5] and costs to health and social care [6]. Declining health and physical limitations may however prevent some older people from taking part in these well evidenced standing programmes. CBE has been developed as a pragmatic way of encouraging exercise for this frailer population providing a more realistically achievable form of exercise [7]. However, until recently, there was no clear definition of what CBE included or where it should be used.

CBE is currently used in a variety of settings such as care homes and day centres [8]; however, there is little robust evidence to suggest that it provides significant health benefits [7]. A systematic review on the benefits of CBE identified a small body of literature with inconclusive findings which offered little guidance for clinical practice [7]. Older people taking part in CBE groups reportedly participate in CBE for a range of reasons including physical and mental health, providing socialisation and friendship, and improving confidence [9]. Few barriers have been identified even amongst older people with physical limitations. This suggests that CBE may be an acceptable and accessible form of exercise for older people with physical limitations who may not be able to take part in other exercise programmes.

A lack of consensus on the fundamental principles of CBE has been highlighted, with the literature describing different interventions delivered in a range of settings and with a different focus [7]. In the absence of clear guidance, a consensus development process was developed to determine the principles of CBE to provide a framework for practice and further research [10]. Through a Delphi study, experts agreed on a definition of CBE and a set of principles providing a clearer underpinning and rationale for programmes [11]. Experts agreed that CBE should contain components of progressive resistance training, cardiovascular interval training, endurance training, and developmental stretches. The potential benefits from the perspective of experts include improving mood and wellbeing, muscle strength, activities of daily living, and joint mobility. Based on the expert consensus, CBE can be defined as “*a primarily seated, structured, and progressive exercise programme that is part of a continuum of exercise for older people, which uses a chair to provide stability, and is delivered by instructors that are suitably skilled and trained to work with frail older people*” [11]. Experts identified that CBE should be used for older people who are unable to take part in other forms of exercise due to activity limitation which may be acute (e.g., following an operation) or of longer term.

The view of experts in the field of exercise for older people and older people taking part in CBE programmes identified the potential benefits of the programmes. However, we know little about the context of delivering CBE or how many instructors are delivering it. Exercise instructors can give us an insight into the types of classes they deliver to older people and the different benefits they observe in older adults in different contexts helping us to further understand when CBE should be used from their perspective. An important consideration in the delivery of exercise programmes is the role of the exercise instructor or leader. Instructors' attitudes and behaviours towards older people's participation in exercise classes have been shown to be influenced by the instructors' qualifications. Those with EXTEND (chair based exercise training provider) exercise qualifications have been found to have more positive attitudes towards older adults participation in mostly seated exercise classes (and therefore CBE) [12]. This suggests that exercise qualification could give instructors a different perspective on the benefits of the classes they deliver [12].

The aim of this paper is to explore instructor use and experiences of delivering mostly seated exercise classes and to consider the findings in relation to the expert consensus on CBE.

## 2. Methods

We carried out a cross-sectional survey with 731 UK exercise instructors with specialist older adult exercise qualifications (Level 3 older adults qualification; see [12]). This is the minimum level of qualification that health services and local councils expect from exercise instructors to be able to deliver to older adults in the United Kingdom. We took a total enumeration approach and tried to reach all exercise instructors with a Level 3 or more qualification. We estimate that at the time of the survey there were some 3,000 older adult instructors with a valid Level 3 qualification in the United Kingdom. In recruiting 731 instructors, we estimate that approximately a quarter of instructors trained and a third of instructors actively delivering exercise programmes with Level 3 qualification participated [12]. This survey investigated instructors' characteristics and attitudes in relation to older adults' participation in exercise classes.

We explored instructors' perceptions of mostly seated exercise classes (classes in which >25% of the time was spent seated were classed as mostly seated) (see [10] for further details), as this fits the definition of CBE in the literature [11]. We asked instructors about the types of classes they delivered and where they delivered these classes. We also asked them about their exercise qualifications (Tables 1 and 2). We asked instructors to report their attitudes to older adults' participation in mostly seated classes using closed questions [12]. These questions were based on the Theory of Planned Behaviour (TPB) [13] and asked questions about instructors' experiences of mostly seated exercise classes. These questions related to whether instructors thought older people could do the exercises (self-efficacy/perceived behavioural control), whether they thought there would be positive or negative outcomes (outcome attitudes) from participation, and whether they thought others influenced older adults participation (social influences). There was also an additional question about whether instructors thought older people would identify the class as relevant to them [12]. Instructors were encouraged to use the free text boxes to provide further context and explanations about their response to the statements (the statements had Likert scale options from “strongly agree” to “strongly disagree”) and to share their general experience of delivering mostly seated classes. Data from the free text comments boxes are used for this study.

Descriptive statistics were used to describe the sample using SPSS Release 22. Framework analysis was used to analyse the qualitative data. Framework analysis was developed through social policy research and can be considered a thematic analytical approach that provides a structured output [14]. The framework for analysis was developed based on the domains from the Delphi study [11]. The domains of “defining CBE,” “intended users,” and “potential benefits” were selected for the analysis framework following initial open coding of a sample of data. Two researchers checked the initial coding and returned to the data to ensure the framework was appropriate. This was then discussed and then the rest of the transcripts were coded using the agreed codes [15]. This was carried out by one researcher (HHH) and then checked independently for agreement by a second researcher (KR). Ethical approval for the questionnaire was granted by the University of Manchester Committee on the Ethics of Research on Human Beings.

## 3. Results

**Figure 1:**
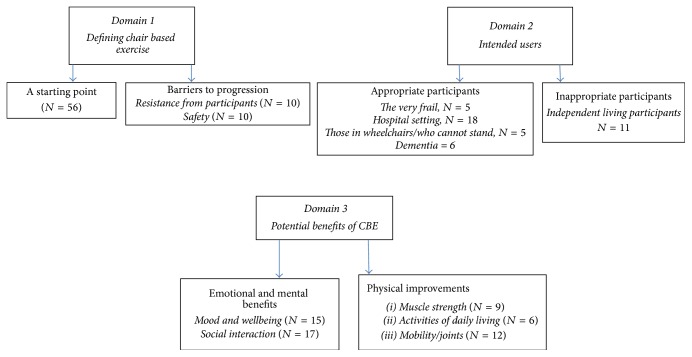
Themes (*N* = the number of times the theme occurred).

378 participants delivered mostly seated exercise classes; age ranged from 22 to 90; 329 (87%) participants were women; a subsample of 223 instructors chose to provide free text responses, with 155 instructors choosing to provide no qualitative comments. In terms of location, the classes were delivered in a range of different settings indicating that the delivery of mostly seated exercise is widespread across a range of populations (Table 2). Instructors who delivered mostly seated exercise classes (and therefore CBE) also had a variety of different qualifications, often multiple qualifications (Table 3). In both tables, information is displayed for the full sample of instructors and the subsample who gave free text responses to give an indication of the broad depth of qualification of instructors and delivery settings for those delivering mostly seated classes. The qualitative themes arising from the survey (Figure 1) are reported under each relevant domain from the CBE Delphi consensus [11] and from this point mostly seated classes are described as CBE.

## 4. Domain One: Defining CBE

*Delphi Statement*. CBE should be considered as part of a continuum of exercise for frail older people where progress is encouraged.

Instructors explored why they delivered CBE and how it fits within the exercise pathway and classes provided. Firstly, they talked about using CBE as a starting point for older people, a place where older people could be introduced to exercise: “I think mostly seated exercise groups are good to introduce patients into exercise” (male, aged 29, PSI Instructor). It could then be used as part of a pathway to other more active classes: “Our rehabilitation level class is viewed as a stepping stone for patients to improve to a level where participation in a ‘prehab' level or LLT structure class can occur” (female, aged 33, PSI Physiotherapist). Instructors used a variety of techniques to progress exercises in a CBE class and to make it more challenging. They encouraged their participants to stand using the chair for support: “we do encourage exercise behind chairs and perhaps holding hands in circles, and so forth to boost balance confidence” (female, aged 77, EXTEND).

However, there were barriers to progressing exercises within the classes and some older people were happy to remain in a CBE class and remain predominantly seated: “I expected to get greater desire for progression but found that in some schemes the people who came were focussed on maintaining low levels of activity” (female, aged 60, PSI Instructor, Occupational Therapist). Sometimes, CBE was delivered for safety reasons; this was because participants needed more support to stand: “I have a few participants who could well benefit from ambulatory exercise, BUT they need one-to-one carer support to help them and keep them safe” (female, aged 74, EXTEND). It could also be too challenging for the instructor to deliver a class to a range of different participants with differing needs: “it is too complex to roll out standing and seated in the same class” (female, aged 51, EXTEND).

## 5. Domain Two: Intended Users

*Delphi Statement*. This is used with older people with an activity limitation who cannot participate in other forms of exercise.

Instructors were very much in agreement with this statement when discussing who they felt CBE classes were most appropriate for. They suggested that CBE classes were appropriate for the very frail: “The majority of my clients are quite frail both physically and mentally and though exercises are important to them, I believe that the mental stimulation and interaction with other people is equally important” (female, aged 63, EXTEND). When delivering exercise to very frail people, they suggested a slightly different approach which also focussed on meeting cognitive needs as well as physical needs.

CBE was found to be particularly useful in a hospital setting: “I use the mostly seated exercise group in an acute elderly ward” (female, aged 49, Physiotherapist, PSI). Instructors also delivered CBE classes with wheelchair users and people with disabilities who could not stand: “I prefer to do seated exercise as I have a lot of service users with different disabilities and some are in wheelchairs…my service users seem to enjoy and feel safe with seated” (female, aged 51, EXTEND). Instructors said that they delivered CBE to those with dementia: “My classes are mainly held during our day centre setting for people living with dementia” (female, aged 66, EXTEND).

They felt that CBE provided them with the opportunity to use a variety of different equipment and approaches to engage people and this was a particularly important tool to engage those with dementia: “My class is 60–90 (years) mostly suffering from dementia, we exercise, we sing, they talk and they like using the equipment. Balls, scarves, battons* [sic]* and strength bands. We dance” (female, aged 67, EXTEND). They also delivered CBE in long-term care and said that although the older people attending the class could not stand there were still benefits to their upper body: “In residential homes where the clients are mainly seated it is essential to keep the upper body as mobile and strong as possible, I taught 2 ladies in wheelchairs and they came for the upper body work” (female, aged 44, EXTEND).

Not only did instructors talk about who CBE was suitable for, but also they discussed who they felt it was not suitable for and how older people did not always benefit from a CBE class. Instructors felt that local council's and health services could encourage older people to participate in CBE even though they should be accessing something which was more challenging: “Some mostly seated classes are delivered because the organisers think that is the appropriate level for older adults exercise. This even happens at Borough Council level. I am constantly explaining to those who should know better that this type of class is not appropriate/should not be first choice for those who live independently and have to manage on their feet on a day to day basis” (male, aged 51, YMCA and PSI).

At times, instructors said that older people were offered CBE for perceived safety reasons or ignorance of the evidence base even though they would get more from a more challenging and active class.

## 6. Domain Three: Potential Benefits of CBE

*Delphi Statement*. If tailored appropriately, CBE can be beneficial in improving the following:

*Emotional and Mental Improvements*

*Mood and wellbeing*
*Social interaction*

Instructors acknowledged that CBE would not always bring condition specific benefits to older adults who had certain conditions, for example, those who were at risk of falls as it could not challenge balance and for those with heart problems requiring more intensive aerobic exercise. However, they did point out the important benefits that the classes could have for older people, in particular for mood and wellbeing across both community settings and long-term care. This was based on self-report from older people in the classes and observed improvements in participants by instructors. Instructors did not report that they routinely carried out formal assessments of function. There was a great emphasis on fun: “We try to make sure that all participants have exercised their laughter muscles” (male, aged 55, YMCA). However, instructors said that they thought this fun also provided older people with the opportunity to improve coordination and memory: “we develop hand-eye-co-ordination and memory skills using agility ladder(s) on floor occasionally; and with ball passing games which have rapidly become one of the favourite parts of the classes” (male, aged 55, YMCA). Linked to mood and wellbeing was the opportunity the classes gave for social interaction. This was seen by instructors as a very important element of CBE classes: “This can be the highlight of someone's week attending and socialising in your chair sessions” (female, aged 48, YMCA/YFIT).

*Physical Improvements*

- *Muscle strength *
- *Certain activities of daily living *
- *Certain personal activities of daily living *
- *Mobility around joints *

Instructors did say that participants could gain physical benefits and improvements from CBE as well as emotional and mental benefits: “As an observation the benefits are improved range of movement (ROM's), social interaction, mental stimulation and general health benefits. Improved strength, stamina and progression of individuals” (female, aged 47, EXTEND).

They mentioned CBE as a way to physically build up to more challenging exercises: “if the individual cannot spend close to an hour standing and exercising, then this allows them to build up strength and stamina” (female, aged 33, PSI and CBE). They talked about how CBE could help older adults with activities of daily living: “Seated exercises can actually make a difference to people's lives - for example, getting into and out of the bath becomes easier” (female, aged 45, YMCA, CBE and Otago). They also reported feedback from older people about the things that they could now achieve since they started attending the class. Even those who could not stand at all or do any leg work could still improve their mobility and ease the pain in their joints: “many attend for other reasons including shoulder/trunk mobility and a good class will provide these things as well…” (female, aged 44, EXTEND). CBE could be particularly helpful for arthritis in the joints: “especially the hips, ankles and the fingers, I've seen remarkable progression with the fingers” (female, aged 46, Health Professional, EXTEND). Instructors pointed out that sometimes a more active class could overlook the smaller joints such as those in the fingers which could get very painful. CBE classes could include a particular focus on joint mobility.

## 7. Discussion

To our knowledge, this is the first exploration of instructor perspectives and expert views on CBE. We present data from instructors delivering exercise programmes in a wide range of settings and with a wide range of qualifications suggesting that CBE is delivered in a variety of different settings to different types of participants regardless of training. There are specific CBE training programmes, but then there are also a wide range of other training programmes which include CBE.

The importance of progressive exercise programmes for older people identified by both experts and instructors is supported by the principles and physiology of exercise for older people [16]. Previous work exploring the attitudes of exercise instructors in this sample identified differences between qualification and attitudes and instructors with an EXTEND qualification demonstrated a more positive attitude towards seated programmes [12]. However, the qualitative feedback provided in this part of the study suggests that there is consensus across most instructors about the delivery of CBE regardless of training or background.

The importance of progression from seated to standing programmes was stressed by instructors and experts. CBE has the potential to introduce older people gently to exercise and increase their motivation and self-efficacy. However, challenges to progression were raised by instructors working with frail older people and there was a consensus across instructors that the class should be tailored to the individual. Previous research looking at both general and condition specific exercise delivery suggests that provision should be person centred and adapted to the individual [17, 18]. It is important that training providers of CBE courses or programmes which include CBE equip their instructors to progress participants and that exercise service providers ensure that a continuum of exercise is provided to enable progression.

Instructors raised issues with CBE being considered a safe, default choice of exercise for older people when it may not be appropriate. This was echoed by experts who identified that CBE should be used for older people with an activity limitation who are unable to take part in standing programmes [11]. Primary research evaluating the benefits of CBE has on occasion included independent community dwelling older people [19] who are not the target population of the intervention which may limit the findings for older people unable to take part in standing programmes.

The benefits of CBE have been suggested to encompass both physical and mental health by instructors and experts. It is interesting that it was predominantly nonclinical instructors who discussed the social and mental benefits of CBE. The reasons for this should be further explored but previous research suggests that it could be because health professionals are focused on more objective functional outcomes and have more limited time to facilitate interaction [12, 20]. Published evidence for CBE is limited and demonstrates a lack of significant benefits of programmes; however, outcomes encompass physical and mental health [7] and suggest that CBE is delivered in a range of settings for a variety of reasons. Our study supports this finding and illustrates the range of different settings CBE is delivered in.

When considering the evidence for CBE, it is important to note the differences between robust trial evidence and qualitative studies and how data from trials at times contradicts instructor's reported experiences. Instructors identified a range of benefits they had observed and their participants had reported as a result of CBE classes. However, there is a lack of clear evidence from randomised controlled trials and other quantitative study designs to support these experiences and instructors did not report objectively measuring outcomes, for example, through functional assessment such as Timed Up and Go [21]. Instructors and experts suggested potential improvements in activities of daily living which is supported in the findings reported by McMurdo and Rennie [22] and Venturelli et al. [23]. Contradictory evidence is however reported in other studies of CBE [24–26] with no significant improvements in activities of daily living reported. There is contradictory evidence to support improvements in muscle strength following CBE with significant improvements reported in care home populations [27] and community dwelling older women [28]. In contrast, a larger well-conducted randomised controlled trial by Latham et al. [26] identified no significant improvement in muscle strength following a seated progressive resistance exercise programme. The lack of clarity over the outcomes of CBE is an area that warrants further consideration. The reduction of pain was identified by instructors in this exploration which was not supported by the expert views. There is a lack of evidence for CBE and the management of pain with no RCTs identified addressing this outcome and other quantitative study designs reporting no significant reduction in pain following CBE [29].

Instructors suggested that CBE is particularly beneficial for those who are frail and particularly the long-term care setting. However, there has been some argument that exercises need to continue to be challenging or they can actually increase older person's chance of a fall [30]. We therefore need to be careful offering only CBE to all frailer older people and older people in long-term care. Previous studies illustrate that, even in the very old, significant improvements in strength can be achieved with quite challenging programmes [31] and this supports the argument that exercises should be tailored and progressive [32].

This is an exploratory study using qualitative data and therefore is limited in the conclusions that can be drawn. In the questionnaire, participants were asked to discuss their mostly seated exercise classes, and the term CBE was not specified. The variation between the levels of standing undertaken in sessions may limit the reliability of the exploration of this study. However, the definition of mostly seated exercise classes fits within the definition of CBE. Defining CBE has been identified as a challenge in both the survey and the Delphi study.

Instructors with a Level 3 older adults exercise qualification were recruited to the study because this is the level acceptable to health services and local councils. However, there are some instructors who may deliver a very prescriptive CBE programme with a Level 2 qualification who could have been excluded from the study and their experiences could differ. It is also possible that the respondents who completed the questionnaire but did not express comments had different views from those who did (e.g., there were no instructors in our responding sample who delivered exercise programmes in NHS venues), and this has to be taken into consideration. However, we do believe that the data does present a wide range of views.

We acknowledge that some of the findings from this work are exploratory and are based primarily on qualitative exploratory research. Although this study adds to our understanding of CBE, the methods cannot objectively determine the health benefits of CBE which would need to be evaluated through a robust randomised controlled trial design.

## 8. Conclusion

Instructors' perspectives largely support the expert consensus on CBE. CBE has an important role to play in a continuum of exercise and may be more suitable for frailer older people. However, it is important that all exercise delivered is tailored and CBE may need to be delivered by experienced instructors or therapists to ensure that it can be delivered at the appropriate level for the individual. From an instructors perspective, CBE has important social benefits even if for some populations the physical benefits are more limited. Future studies are required to further establish the benefits of CBE and its appropriate use.

*Recommendations*

- CBE should not be seen as a default option for all older adults and instead should be appropriately targeted for older adults that are unable to take part in standing programmes.
- Progression within programmes should be encouraged and tailored to individual need.
- Training providers of qualifications in CBE or programmes including CBE should ensure instructors/therapists have the skills and confidence to progress their exercise participants
- Organisations offering CBE classes should also provide further exercise opportunities for older adults where they can progress.
- There is a requirement for well-designed studies to explore the physical, mental, and social benefits of CBE in a range of different older populations.

## Figures and Tables

**Table 1:** Explanations of training qualifications.

Qualifications	Description^*∗*^
EXTEND	Provides gentle movement to music for older people and for anyone of any age with a disability.
Later Life Training, Postural Stability Instructor (PSI)	Provides a range of professionals with the skills to deliver effective and fun exercise opportunities, which include strength and balance exercises for older people with a fear or history of falls.
YMCA/YFIT	Can specialise in exercise to music, CBE, weights, or circuit training suitable for older adults.
Later Life Training, Otago Exercise Programme Leader	Provides evidence based home exercise and small group exercise options based on strength and balance exercises to prevent falls and injuries and improve cognition amongst older people.
Later Life Training, Chair Based Exercise Leaders (CBE)	CBE Programme for Older Adults and Disabled Older Adults
Fitness League	Training as a Fitness League teacher will provide you with a YMCA Award Level 3 Certificate in Teaching Exercise, Movement and Dance and the EMDP (Fitness League) Level 3 Certificate in Teaching Exercise, Movement and Dance to Adults (Bagot Stack). No further information.
KFA	Noncompetitive exercise, movement and dance based sessions. Aimed to enhance daily life and to maintain a good level of posture, mobility, and coordination. Ideal for the active retired.
Medau	Working with a variety of music and rhythms, Medau movement encourages the body to move with energy, strength, stamina, suppleness, and coordination. Focusing on correct posture and body alignment, Medau movement has a natural, flowing quality, whilst at the same time being dynamic, lifting the spirits, and increasing confidence.
Laban	See KFA. KFA based on Laban principles. No other information available.
BACR (L4)	Enables the instructor to safely prescribe and deliver an exercise programme for individuals with cardiovascular disease.

^*∗*^Description provided by the provider.

**Table 2:** Type and location of classes.

Type and location	Number of instructors, full sample, *N* = 378	Number of instructors, subsample, *N* = 223 (%)
50% seated classes	168 (44.4%)	73 (32.7%)
75% seated classes	161 (42.6%)	65 (29.1%)
Fully seated classes	174 (46%)	75 (33.6%)
Community venues	191 (50.5%)	113 (50.7%)
Leisure centre/gym	37 (9.8%)	18 (8.1%)
Sheltered housing	113 (29.9%)	50 (22.4%)
Residential home	92 (24.3%)	34 (15.2%)
Nursing home	48 (12.7%)	29 (13%)
EMI home^*∗*^	34 (9%)	24 (10.8%)
National Health Service (NHS) venue	44 (11.6%)	0 (0%)
Day centre	21 (5.5%)	0 (0%)

^*∗*^Elderly mentally infirm.

*Note.* Instructors could deliver more than one type of class in more than one location.

**Table 3:** Instructors training qualifications.

Instructors training	Number of instructors, *N* = 378^*∗*^	Number of instructors, subsample, *N* = 223 (%)
EXTEND	237 (62.7%)	149 (66.8%)
Later Life Training, Postural Stability Instructor (PSI)	82 (21.7%)	41 (18.4%)
YMCA/YFIT	28 (7.4%)	15 (6.7%)
Later Life Training, Otago Exercise Programme Leader	29 (7.7%)	14 (6.3%)
Later Life Training, Chair Based Exercise Leaders (CBE)	29 (7.7%)	8 (3.6%)
Fitness League	31 (8.2%)	7 (3%)
KFA	13 (3.4%)	9 (4%)
Medau	3 (0.8%)	2 (1%)
Laban	2 (0.5%)	1 (0.5%)
BACR (L4)	9 (2.4%)	2 (1%)

^*∗*^Most instructors had multiple qualifications.

## Abbreviations

CBE: Chair based exercise.
