# Corrigendum to “Instructors' Perceptions of Mostly Seated Exercise Classes: Exploring the Concept of Chair Based Exercise”

**DOI:** 10.1155/2017/1868251

**Published:** 2017-05-14

**Authors:** Katie R. Robinson, Tahir Masud, Helen Hawley-Hague

**Affiliations:** ^1^Division of Rehabilitation and Ageing, University of Nottingham, Queen's Medical Centre, Nottingham NG7 2UH, UK; ^2^Bassetlaw Health Partnership, Nottinghamshire Healthcare NHS Foundation Trust, Day Rehabilitation, Retford Primary Care Centre, North Road, Retford DN22 7XF, UK; ^3^Healthcare of Older People, Nottingham University Hospitals NHS Trust, Queen's Medical Centre, Nottingham NG7 2UH, UK; ^4^Nursing, Midwifery and Social Work, The University of Manchester, Jean McFarlane Building, Floor 6, Room 332, Oxford Road, Manchester M13 9PL, UK

In the article titled “Instructors' Perceptions of Mostly Seated Exercise Classes: Exploring the Concept of Chair Based Exercise” [1], there was an error in the Acknowledgments, which should be corrected as follows:

This work was supported by the Medical Research Council (MRC) Doctoral Training Grant (Grant no. MR/K500823/1) and the University of Manchester through the Faculty of Medical and Human Sciences. The authors would also like to thank Professor Dawn Skelton for her advice and Professor Chris Todd, Professor Skelton, and Dr. Maria Horne for their involvement in the original survey design.
